# Expansion of Polymorphonuclear Myeloid-Derived Suppressor Cells in Patients With Gout

**DOI:** 10.3389/fimmu.2020.567783

**Published:** 2020-10-14

**Authors:** Limei Zhong, Sitao Li, Yi Wen, Junhui Zheng, Fengbin Liu, Donglin Cao, Yufeng Liu

**Affiliations:** ^1^Department of Laboratory Medicine, Guangdong Second Provincial General Hospital, Guangzhou, China; ^2^Department of Neonatology, The Sixth Affiliated Hospital of Sun Yat-sen University, Guangzhou, China; ^3^The First Affiliated Hospital, Guangzhou University of Chinese Medicine, Guangzhou, China; ^4^Guangzhou First People's Hospital, Guangzhou, China

**Keywords:** gout, immunosuppressive, T cell function, monosodium urate crystals, Polymorphonuclear myeloid-derived suppressor cell

## Abstract

Gout is an inflammatory joint disease caused by monosodium urate (MSU) crystals; however, the mechanism underlying MSU-induced inflammation is unclear. Previous research has suggested that inflammation or cancer can drive the expansion of myeloid-derived suppressor cells (MDSCs). In this study, the role of MDSCs in MSU-induced gout inflammation was evaluated. A total of 28 patients with gout, and 20 healthy controls were recruited for the study. MDSCs, and their functions, were analyzed by flow cytometry and a T cell co-culture assay, respectively. We observed a higher frequency of PMN-MDSCs, and a stronger immunosuppressive function, in patients with gout compared to the controls. Moreover, circulating PMN-MDSCs were positively correlated with pathological indicators, including uric acid and C-reactive protein levels. We also demonstrated that MSU can induce significant PMN-MDSC expansion, using *in vivo* and *in vitro* experiments. Finally, MSU-induced PMN-MDSCs produced higher levels of IL-1β, which mediated gout inflammatory progression. Our results demonstrate that MSU modulates the expansion and suppressive function of PMN-MDSCs, providing insights into a novel mechanism underlying the pathogenesis of MSU-induced gout. Thus, MDSCs may be useful for the development of novel therapeutic strategies for the prevention and treatment of gout.

## Introduction

Gout is defined as acute aseptic inflammation caused by the inflammatory response of joint tissues to monosodium urate crystals (MSU) (1). As an increasingly serious arthritic disease, particularly in China, gout has become an important health issue (2). However, the mechanisms underlying the development of gout are complex. Multiple innate immune cells are involved in the pathogenesis of acute gout inflammation, such as monocytes, macrophages, mast cells, neutrophils, and NK cells (3–5). Meanwhile, MSU crystals are considered the “culprits” behind the immune response, rather than simply waste products of purine catabolism (6, 7). Similar to microorganisms, MSU crystals serve as dangerous signals, causing a proinflammatory response after they are taken up by macrophages leading to activation of NALP3 (also known as NLRP3) inflammatory bodies, and subsequent secretion of the proinflammatory cytokines interleukin (IL)-1β and IL-18, and recruitment of neutrophils (8, 9). Neutrophils also take up MSU crystals and initially respond by releasing inflammatory mediators, including tumor necrosis factor (TNF), IL-6, and IL-8 (10). Although MSU is a well-established cause of gout, the detailed mechanisms underlying the inflammatory process are not fully understood. Furthermore, the mechanism for rapidly controlling and terminating MSU-induced inflammation is unclear.

Myeloid-derived suppressor cells (MDSCs) are a heterogeneous cell population with important regulatory functions, primarily based on the inhibition of T cell proliferation (11–13). Human MDSCs are CD11b^+^CD33^+^HLA-DR^−^ and can be further classified into two major subsets, CD11b^+^CD33^++^ monocytic MDSCs (M-MDSCs), and CD11b^+^CD33^+^ polymorphonuclear MDSCs (PMN-MDSCs) (14, 15). Murine MDSCs are characterized by the co-expression of Gr-1 and CD11b and can be further subdivided into CD11b^+^Gr1^+^Ly6C^+^ M-MDSCs and CD11b^+^Gr1^+^Ly6G^+^ PMN-MDSCs (16, 17). MDSC accumulation and activation increase the production of reactive oxygen species (ROS), arginase 1 (Arg-1), and nitric oxide (NO) in secondary lymphoid tissues, and inhibit the immune responses of CD4 T cells, CD8 T, cells and NK cells (18). Note, the heterogeneity of MDSCs indicates that there may not be a unique phenotypic marker that can accurately represent MDSCs, and thus, their specific inhibitory activity may serve as their ultimate defining characteristic (19).

Currently, the role of MDSCs in various inflammatory diseases remains controversial. For instance, some studies suggest that MDSCs can break the vicious cycle of inflammation and autoimmunity, delay the process of autoimmune arthritis, and improve rheumatoid arthritis (RA) (20, 21). While another study reported that MDSCs become significantly expanded in an arthritic mouse model, and in RA patients, and produce high levels of inflammatory cytokines. Alternatively, MDSCs from a collagen-induced arthritis mouse model, as well as RA patients, have been shown to promote the polarization of Th17 cells *in vitro*, while significantly inhibiting the ability of T cells (22). Hence, MDSCs have been identified as therapeutic targets for many rheumatic diseases, such as RA and systemic lupus erythematosus (23, 24). However, it remains unclear whether MDSCs and their subgroups, were abnormally expressed in patients with gout.

In this study, we observed that the frequency of PMN-MDSCs was significantly higher in the peripheral blood of patients with gout compared to controls. We also defined the correlation between PMN-MDSCs and disease activity. Moreover, MSU was found to induce PMN-MDSC expansion and confer its T cell suppression function *in vivo* and *in vitro*. Finally, mechanistic studies showed that MSU induces MDSCs to secrete IL-1β, which contributes to the gout inflammatory response. The mechanisms established in our study provide a basis for effectively interfering with the inflammatory pathway in gout, and for the development of an effective MDSC-targeted cellular immunotherapy.

## Materials and Methods

### Patients

Patients with gout [determined by the 1977 ARA preliminary criteria (25)] and healthy donors were recruited from Guangdong Second Provincial General Hospital between September 2018 and October 2019. Detailed patient characteristics are listed in Table 1. The study was approved by the Clinical Ethics Review Board of the Guangdong Second Provincial General Hospital. Written informed consent was obtained from all patients at the time of admission.

**Table 1:** Characteristics of the patients and controls included in the study.

**Variable**	**Gout patients *N* = 28**	**HCs *N* = 20**
Age, y	43.9 ± 5.5	41.9 ± 6.1
Gender (Male)	13 (46.4%)	11 (55%)
Disease duration, y	2.1 ± 0.95	Not available
Tophi	7 (25%)	Not available
Deformity	3 (10.7%)	Not available
CRP, mg/L	10.6 ± 19.74	5.6 ± 2.2
Urate, mmol/L	439.5 ± 105.943	225 ± 95.68
**Medication**		
NSAIDs (*n*)	10	Not available
Colchicine (*n*)	11	Not available
Prednisone (*n*)	2	Not available
Allopurinol (*n*)	7	Not available
Benzbromarone (*n*)	5	Not available

*CRP, C-reactive protein; HC, healthy controls; NSAIDs, nonsteroidal anti-inflammatory drugs*.

### Mice

C57BL/6 and BALB/c mice were purchased from the experimental animal center of Guangdong Province. All animal experiments were approved by the Animal Care and Ethics Committee of Guangzhou University of Chinese Medicine. Mice were maintained under specific pathogen-free conditions and fed a standard diet. The experimental procedures were performed in accordance with the Guidelines for Animal Experimentation of Guangzhou University of Chinese Medicine (Guangzhou, China).

### T Cell Proliferation Assay

For human samples, MDSCs (including PMN-MDSCs and M-MDSCs) from peripheral blood mononuclear cells (PBMCs) were plated on U-bottomed 96-well plates, co-cultured with autologous CD3^+^ T cells at different ratios (MDSC:T cell, 1:2/4/8), which were labeled with carboxyfluorescein succinimidyl amino ester (CFSE) (3 μM; Invitrogen, Carlsbad, CA, USA), and stimulated with CD3/CD28 antibodies (eBioscience, San Diego, CA, USA). After 72 h, cells were stained with anti-CD4 and anti-CD8a, and T cell proliferation was measured by flow cytometry.

For mouse samples, MDSCs (including PMN-MDSs and M-MDSCs) from MSU-treated bone marrow (BM) cells were plated on U-bottomed 96-well plates, co-cultured with Balb/c mice spleen-derived CD3^+^ T cells at different ratios (MDSC:T cells, 1:2/4/8), which were labeled with CFSE (3 μM), and stimulated with mouse CD3/CD28 antibodies. After 72 h, cells were stained with anti-CD4 and anti-CD8, and T cell proliferation was measured by flow cytometry.

For the inhibitor test, 0.5 mM N-hydroxyl-nor-l-arginine (nor-NOHA) (Cayman Chemicals, Chemicals, Ann Arbor, MI, USA), an arginase I-specific inhibitor; 1 mM N-acetylcysteine (Sigma, Darmstadt, Germany), a ROS inhibitor; and L-NMMA, an iNOS inhibitor, were added to the culture on day 0.

### MSU Crystal Preparation

Urate acid sodium salt (Sigma-Aldrich, St. Louis, MO, USA) was dissolved in 1 M NaOH (25 mg/mL), heated to 70°C, and the pH was adjusted to 7.2–7.4. The solution was left to cool at room temperature and filtered through a 0.22 mM filter.

### Flow Cytometric Analysis

Blood samples were analyzed within 4 h after sampling, and PBMCs were isolated by Ficoll density gradient centrifugation. Antibodies for flow cytometry are listed in Supplementary Table 1; Samples were quantified and analyzed by flow cytometry (BD LSRFortessa; Franklin Lakes, NJ, USA), and data were analyzed using FlowJo, version 10.

### Flow Cytometric Sorting

For flow cytometric sorting, a fluorescence activated cell sorter (FACS) Aria II (BD, Mountain View, CA, USA) was used. The strategy for PMN-MDSC, and M-MDSC sorting was HLA-DR^−/low^CD11b^+^CD33^+/high^ cells from live PBMCs expressing the following markers: PMNs, CD11b^+^HLA-DR^−/low^CD15^+^CD14^−^; MONs: CD11b^+^HLA-DR^−/low^CD14^+^CD15^−^. The gating strategy for mouse MDSCs was: CD11b^+^Ly6C^hi^Ly6G^−^ for M-MDSCs and CD11b^+^Ly6C^−^Ly6G^hi^ for PMN-MDSCs.

### *In vitro* MDSC Induction

MDSC induction was performed as described previously (26). PBMCs were isolated from healthy volunteers by density gradient centrifugation. PBMCs were cultured in complete 1640 medium (supplemented with 10% fetal bovine serum, 1 mM penicillin-streptomycin, and 50 mM 2-mercaptoethanol) for 5 days and supplemented with cytokines, as indicated, including granulocyte-macrophage colony stimulating factor (GM-CSF; 10 ng/mL) and IL-6 (10 ng/mL), and MSU (50 μg/mL, Sigma). Cultures were run in duplicate, and the media and cytokines were refreshed every 2–3 days. For mouse culture, WT mouse (C57BL/6 background) BM cells were isolated, cultured with complete 1640 medium, and supplemented with cytokines, as indicated, including GM-CSF (10 ng/mL), and IL-6 (10 ng/mL), with or without MSU (50 μg/mL).

### Quantitative Polymerase Chain Reaction (PCR)

RNA was extracted using a multisource total RNA miniprep kit (AXYGEN Biosciences, Hangzhou, China) according to manufacturer's instructions. qRT-PCR was performed as described previously. Primers are listed in Supplementary Table 2.

### ROS Measurement

Oxidation-sensitive dye dichlorodihydrofluorescein diacetate (DCFDA, Molecular Probes/Invitrogen) was used to measure ROS production by MDSCs. Cells were incubated at 37°C in Rosewell Park Memorial Institute (RPMI)1640 medium in the presence of 2.5 μM DCFDA for 30 min. Cells were washed with phosphate-buffered saline and analysis was then conducted by flow cytometry.

### Gout Mouse Model

WT mice were anesthetized with 2.5% isoflurane, and MSU (100 μg/10 μL) or vehicle was injected into the right tibiotarsal joint (ankle) of each mouse for 3 d as described previously (27). The proportion of MDSCs in the BM and peripheral blood was analyzed by flow cytometry, and a T cell proliferation assay was used to evaluate the inhibitory function of gout mouse-derived MDSCs.

### T Cell Polarization

Purified CD4^+^CD45RA^+^ naïve T cells were cultured for 6 or 7 d according to specific differentiation conditions for Th1/2/17 and Tregs (28). PMN-MDSCs (at a 1:1 ratio to the naïve CD4 T cells) were added to determine the role of MDSCs in regulating T cell polarization.

### Statistical Analyses

All data are presented as means ± standard deviation (SD). Statistical analyses were performed using unpaired or paired *t*-tests. The Spearman rank test was used to analyze the correlations between parameters. Statistical tests were performed using GraphPad Prism version 8.0 (GraphPad Software, San Diego, CA, USA), and *P*-values < 0.05, 0.01 or 0.001 were considered significant.

## Results

### Clinical Characteristics of Patients With Gout

In total, 28 patients with gout, and 20 age- and sex-matched healthy controls (HCs) were recruited. The urate and C-reactive protein (CRP) levels in patients with gout were significantly higher than those in HCs (*P* < 0.05), as summarized in Table 1.

### Expansion of PMN-MDSC in Disease of Gout

**Figure 1:**
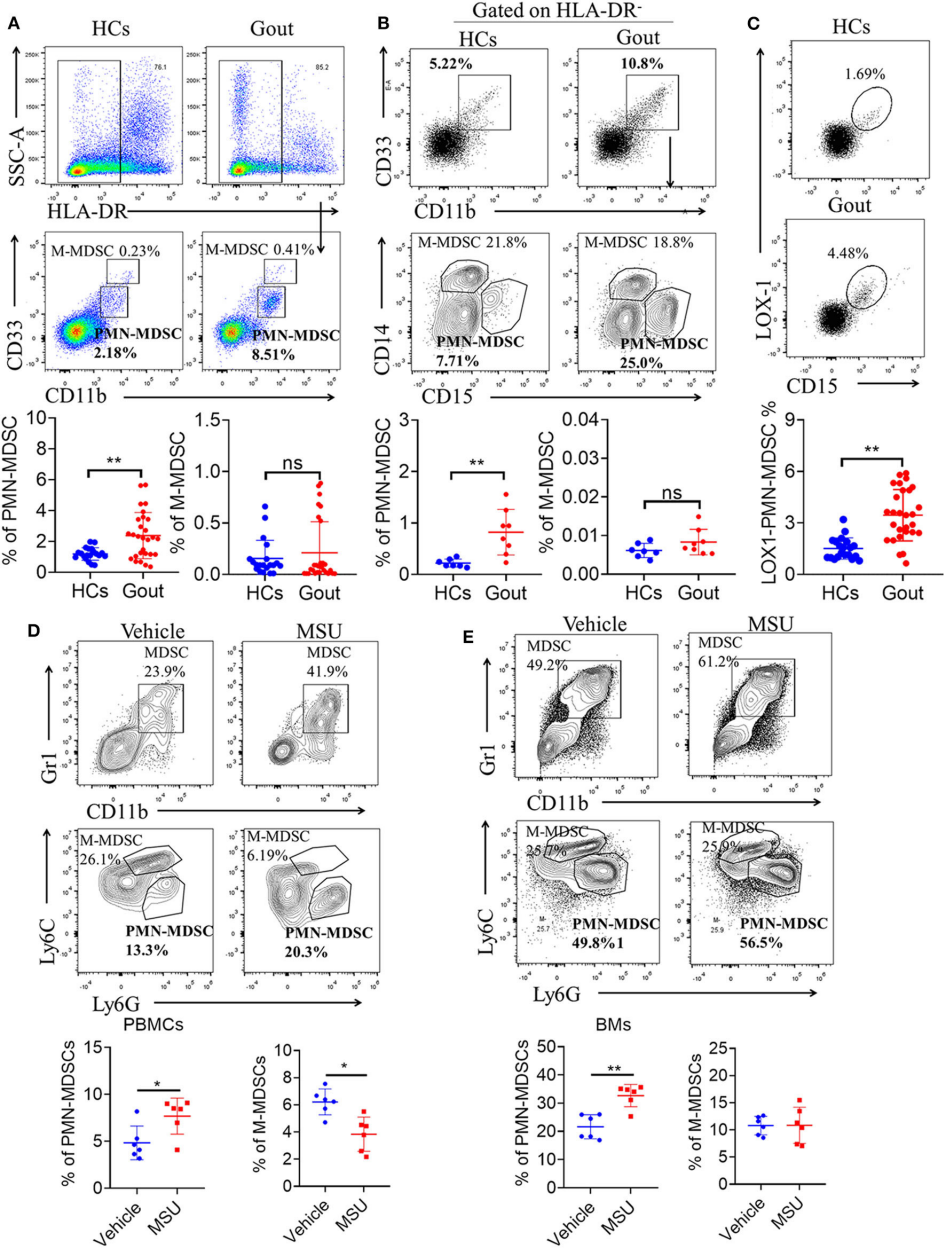
Expansion of PMN-MDSCs in gout disease. **(A)** Representative flow plots and statistical analysis for the proportion of HLA-DR^−^CD33^++^CD11b^+^ cells (M-MDSCs), and HLA-DR^−^CD33^+^CD11b^+^ cells (PMN-MDSCs) in patients with gout (HCs, *n* = 20; gout, *n* = 28); **(B)** Representative flow plots and statistical analysis showing the proportion of HLA-DR^−^CD33^+^CD11b^+^CD14^+^CD15^−^ cells (M-MDSCs), and HLA-DR^−^CD33^+^CD11b^+^ CD14^−^CD15^+^ cells (PMN-MDSCs) in patients with gout (HCs, *n* = 7; gout, *n* = 8); **(C)** Representative flow plots and statistical analysis for the proportion of LOX1^+^CD15^+^-PMN-MDSCs in patients with gout (HCs, *n* = 20; gout, *n* = 28); **(D,E)** Representative flow plots and statistical analysis for the proportion of M-MDSCs (CD11b^+^Gr1^+^Ly6C^+^Ly6G^−^), and PMN-MDSCs (CD11b^+^Gr1^+^Ly6C^−^Ly6G^+^) in PBMCs **(D)** and BMs **(E)** from a gout mouse model (*n* = 6). Error bars represent the mean ± SD; ^*^P < 0.05, ^**^P < 0.01.

The frequency of MDSCs and their subsets from PBMCs of gout patients was determined via flow cytometry. Figure 1A shows the gating strategy and analysis of the two major MDSC subsets based on staining for HLA-DR, CD11b, and CD33. Whole MDSCs (defined as HLA-DR^−^/CD33^+^/CD11b^+^ cells) were measured and quantified in freshly drawn blood from all study subjects. First, HLA-DR-positive cells were excluded and CD33 and CD11b-positive cells were further gated. Two distinct populations were observed: a CD33^++^/CD11b^+^ population, characterized as M-MDSCs and a CD33^+^/CD11b^+^ population, characterized as PMN-MDSCs (Figure 1A). Next, we observed that the frequency of PMN-MDSCs was significantly higher in the peripheral blood of patients with gout (*P* < 0.01) than in controls (Figure 1A). However, the proportion of M-MDSCs did not differ significantly between patients with gout and controls (Figure 1A). In addition, we used CD14 and CD15 to define the population of two subsets of MDSCs and results also showed that CD15 positive PMN-MDSCs were significant expanded in patients with gout compared to the controls (Figure 1B). Lectin-type oxidized low-density lipoprotein receptor-1 (LOX-1) is a unique surface marker of human MDSCs and is the first established surface marker of PMN-MDSCs (29). We also observed that LOX-1^+^CD15^+^ PMN-MDSCs were significantly more frequent in PBMCs of patients with gout than in HCs (Figure 1C). However, there was no difference in the PMN-MDSC population between patients with and without a gout flare (Supplementary Figure 1A). Furthermore, we found that PMN-MDSCs were significantly expanded in the peripheral blood, and BM of mice in the gout model (Figures 1D,E). These results indicated that there is a significant increase in PMN-MDSCs in patients and mice with gout.

### Elevated PMN-MDSCs Correlate With Gout Disease Activity

**Figure 2:**
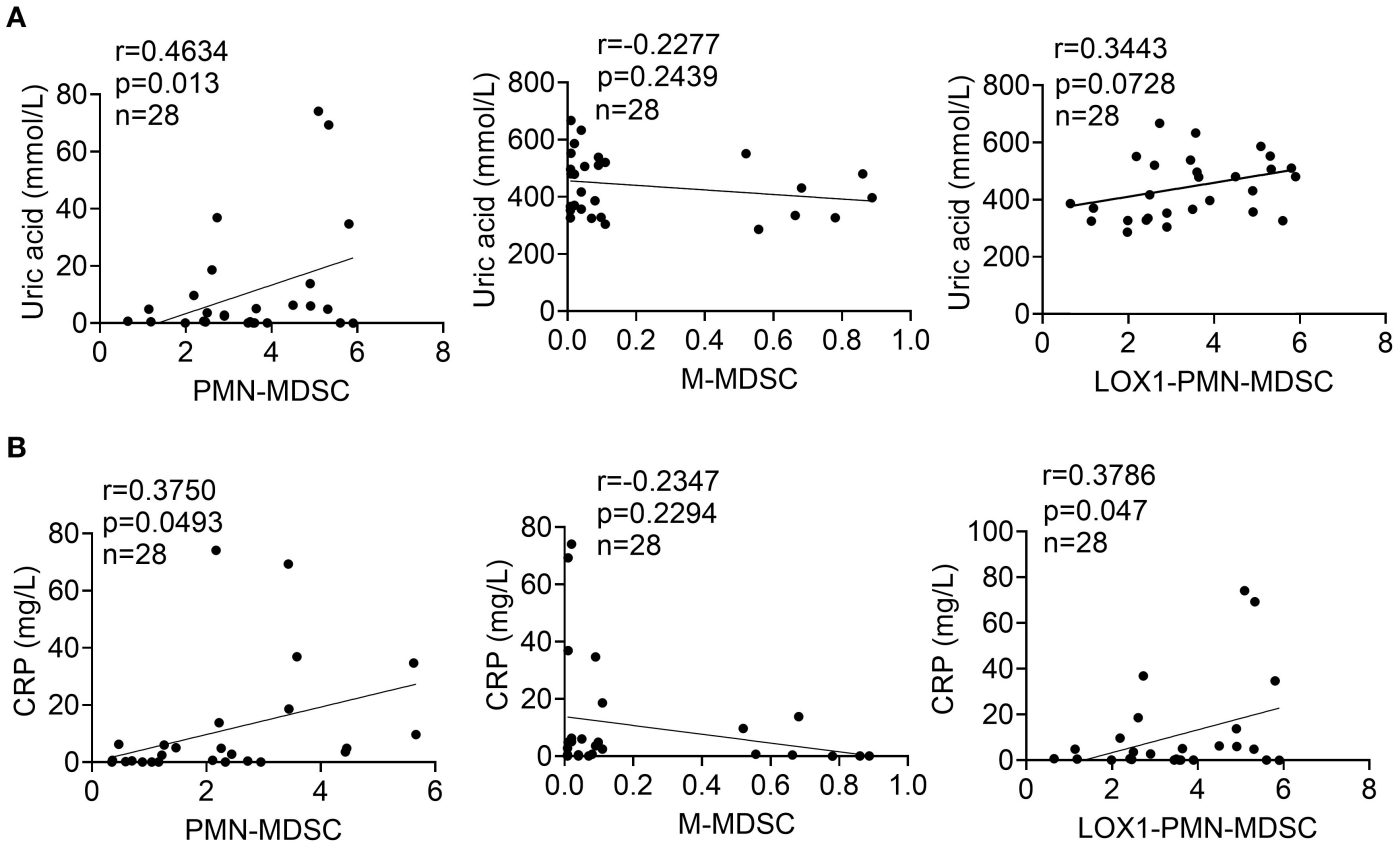
Elevated PMN-MDSCs correlate with gout disease activity. **(A)** Correlations between levels of uric acid and the proportions of circulating PMN-MDSCs, M-MDSCs, and LOX1^+^CD15^+^-PMN-MDSCs in patients with gout; **(B)** Correlations between levels of CRP and the proportions of circulating PMN-MDSCs, M-MDSCs, and LOX1^+^CD15^+^-PMN-MDSCs in patients with gout.

To elucidate the clinical relevance of the increase in circulating PMN-MDSCs, we analyzed whether the frequency of PMN-MDSCs is correlated with physiological parameters related to disease status, including uric acid and CRP levels. We observed that peripheral blood PMN-MDSCs were positively correlated with uric acid levels (*P* < 0.013, *r* = 0.4634) in gout (Figure 2A). However, there was no significant correlation between the frequencies of circulating M-MDSCs and LOX-1^+^CD15^+^ PMN-MDSCs (Figure 2A). Furthermore, increased levels of PMN-MDSCs (*P* < 0.0493, *r* = 0.3750) and LOX-1^+^CD15^+^ PMN-MDSCs (P < 0.047, *r* = 0.3786) were correlated with higher levels of CRP (Figure 2B). These results suggest that PMN-MDSCs are closely related to the disease activity of gout.

### Gout-Derived PMN-MDSCs Suppress T Cell Proliferation via ROS

**Figure 3:**
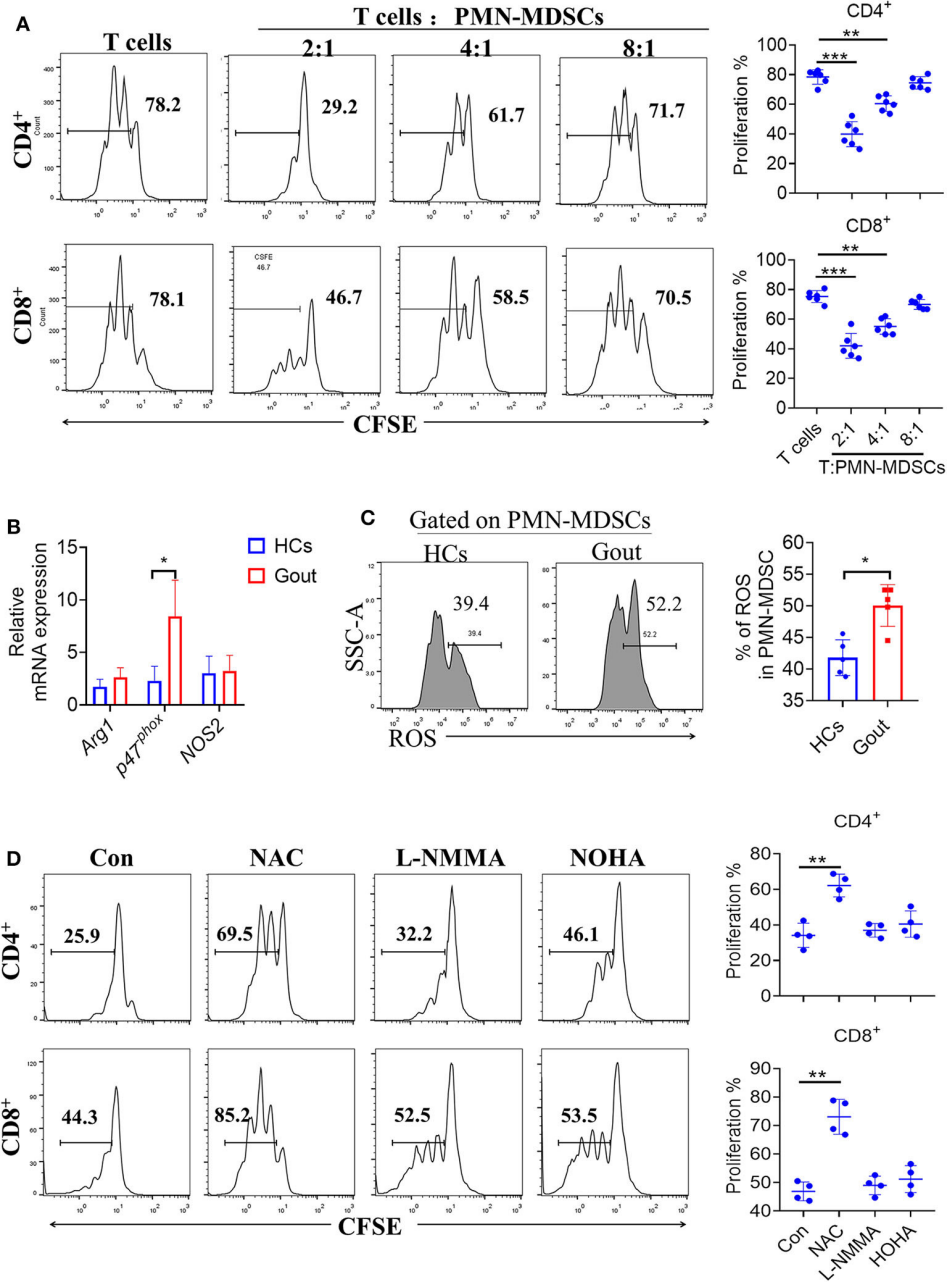
Gout-derived PMN-MDSCs suppress T cell proliferation via ROS. **(A)** Representative histograms for the T cell proliferation assay. PMN-MDSCs sorted from patients with gout and co-cultured with T cells at different ratios (1:2/4/8) (*n* = 6). **(B)** PMN-MDSCs sorted from HCs and patients with gout, qRT-PCR analysis the T cells proliferation suppressed molecules (*n* = 5). **(C)** Representative flow plots and statistical analysis for the levels of ROS in patients with gout or HCs (*n* = 6); **(D)** Effect of different inhibitors on the function of gout-derived PMN-MDSCs was evaluated by T cell proliferation assay as described in **(A)** at a 2:1 ratio for 3 days with treatments as indicated. Unstimulated T cells were used as a negative control. nor-NOHA (100 μM): arginase inhibitor; L-NMMA (100 μM): iNOS inhibitor; NAC (1 mM): ROS inhibitor. Data are representative of two independent experiments. Error bars show mean ± SD; ^*^P < 0.05, ^**^P < 0.01, ^***^P < 0.001.

To confirm the suppression of T cell proliferation by PMN-MDSCs in gout, we isolated and co-cultured CD3/CD28-stimulated T cells with autologous PMN-MDSCs *in vitro*. CFSE-labeled CD3^+^ T cells were cultured with or without PMN-MDSCs over a period of 3 days. CD4^+^ and CD8^+^ T cells showed baseline proliferation rates of 78.2 and 78.1%, respectively, when cultured alone in stimulation media. When autologous PMN-MDSCs were added to these cultures at different ratios of (MDSCs:T cells, 1:2/4/8), CD4^+^ and CD8^+^ T cell proliferation decreased significantly, confirming the suppressive function of MDSCs (Figure 3A). Meanwhile, gout-derived M-MDSCs did not display T cell suppressive function (Supplementary Figure 2A).

Based on the observation that gout-derived PMN-MDSCs suppressed T cell responses, we further explored the underlying mechanisms controlling this suppression. We, therefore, compared the levels of the L-arginine metabolic products: arginase, nitric oxide (NO), and ROS between gout-PMN-MDSCs and control-MDSCs. Results showed that *P47*^*phox*^ was significantly increased in gout-PMN-MDSCs compared to control-PMN-MDSCs, whereas the mRNA levels of *arg-1* and *NOS2* were not significantly affected (Figure 3B). Increased ROS levels were also observed in PMN-MDSCs from patients with gout compared to control-PMN-MDSCs (Figure 3C), these results suggest that ROS plays an essential role in gout-derived PMN-MDSC-mediated immune suppression. Meanwhile, the addition of NAC (ROS inhibitor) to the MDSC-T cell co-culture system caused a near complete abrogation of the suppressive effect on CD4^+^ and CD8^+^ T cells, whereas the inhibitors for arginase and iNOS had no such effect (Figure 3D). Therefore, we concluded that gout-MDSCs suppress T cells in an ROS-dependent manner.

In addition, proliferation of T cells is an important parameter, thus, necessary to examine the polarization of T cells (Th1/2/17 and Treg). To this end, PBMCs were prepared by density gradient centrifugation using Ficoll. Purified CD4^+^CD45RA^+^ naïve T cells were cultured for 6 or 7 d according to specific differentiation conditions for Th1/2/17 and Tregs. PMN-MDSCs were isolated from HCs or gout-derived-PBMCs by cell sorting and PMN-MDSCs (at a 1:1 ratio to the naïve CD4 T cells) were added to determine the role of MDSCs in regulating the polarization of T cells. Results showed that there was no difference in T cell polarization induced by PMN-MDSCs from patients with gout compared to HCs (Supplementary Figure 3A).

### Direct MSU Co-culture Promotes MDSC Expansion

**Figure 4:**
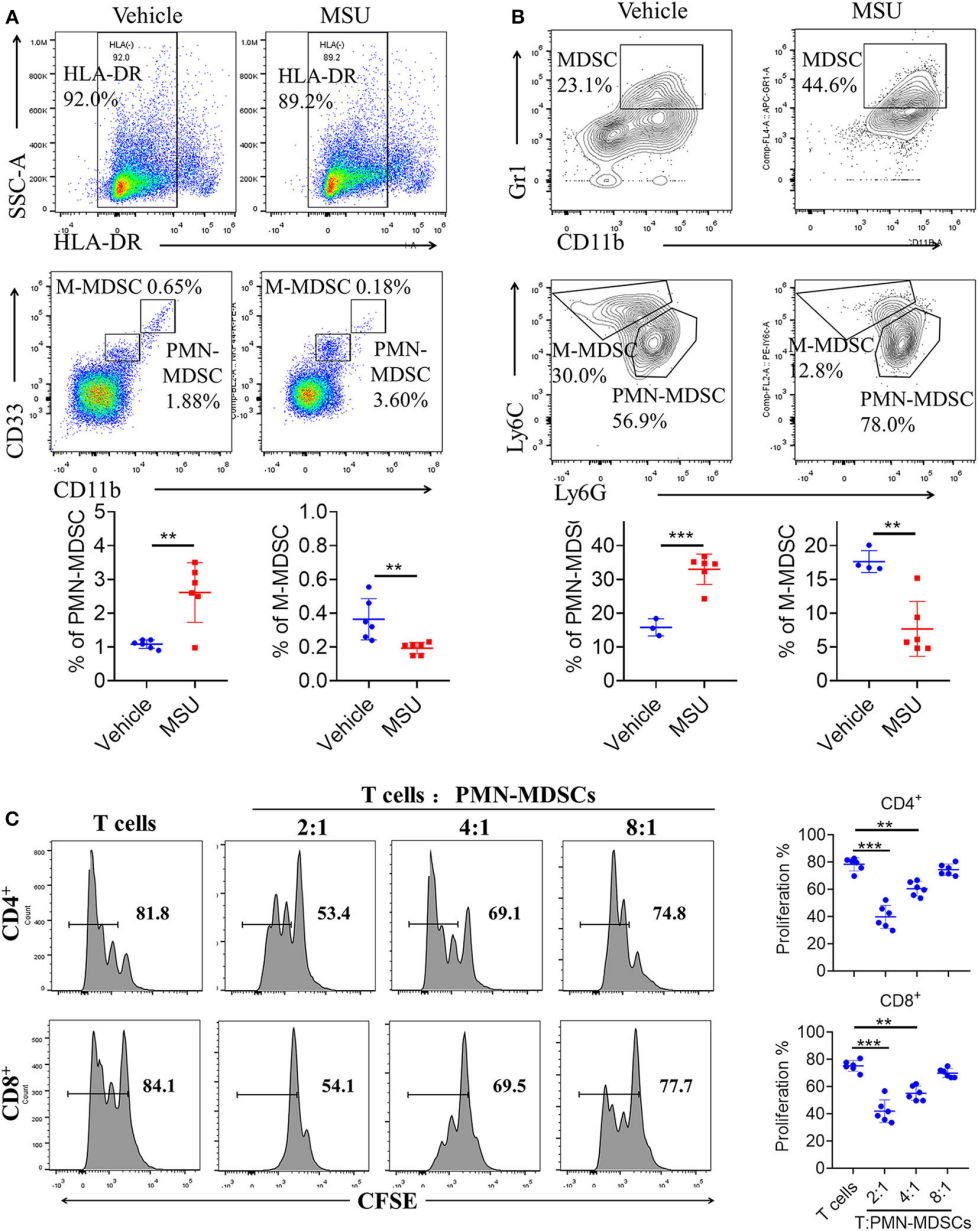
MSU induces high levels of MDSCs *in vitro*. **(A)** Representative flow plots and statistical analysis for the proportion of HLA-DR^−^CD33^++^CD11b^+^ cells (M-MDSCs), and HLA-DR^−^CD33^+^CD11b^+^ cells (PMN-MDSCs) in vehicle- or MSU crystal-treated PBMCs from healthy controls. **(B)** Representative flow plots and statistical analysis for proportions of (CD11b^+^Gr1^+^Ly6C^+^Ly6G^−^) M-MDSCs, and (CD11b^+^Gr1^+^Ly6C^−^Ly6G^+^) PMN-MDSCs in vehicle- or MSU crystal-treated BMs from WT mice. **(C)** Allogeneic mixed lymphocyte reaction. CD3^+^ T cells (from BALB/c mice) were stimulated with CD3/CD28 and co-cultured with allogeneic PMN-MDSCs from MSU crystal-treated BMs for 3 days. T cell proliferation was evaluated by CFSE dilution. Unstimulated T cells were used as a negative control. Three independent experiments with similar results were performed and mean + SD of six samples pooled from three experiments is shown. ^**^P < 0.01, ^***^P < 0.001.

The clinical significance of PMN-MDSCs in the pathogenesis of gout disease prompted us to investigate the mechanisms of MDSC expansion in MSU treatment. First, we explored whether direct MSU co-culture could lead to the accumulation of PMN-MDSCs *in vitro*. GM-CSF has been shown to efficiently induce suppressive MDSCs *in vitro* from the PBMCs of healthy donors or BM from mice. Therefore, we utilized the system to study the effect of MSU on MDSC generation. Healthy donor-derived PBMCs were stimulated with or without MSU and cultured for 5 days in the presence of GM-CSF. Our results show that MSU significantly increased the expansion of PMN-MDSCs *in vitro*; however, the proportion of M-MDSCs significantly decreased (Figure 4A). To further evaluate the effect of MSU on mouse MDSCs, mouse BM cells were cultured in medium containing GM-CSF with or without MSU to generate MDSCs *in vitro*; We found that MSU consistently expanded the population of PMN-MDSCs, but not of M-MDSCs (Figure 4B). The effect on MSU on the induction of PMN-MDSCs expansion, we found that functional studies further verified that PMN-MDSCs generated *in vitro* were immunosuppressive to T cells (Figure 4C).

Finally, to determine whether transfer of MSU-induced PMN-MDSCs can aggravate mouse gout symptoms, we performed *in vivo* transfer of MSU-induced PMN-MDSCs (Supplementary Figure 4A). Compared to vehicle-induced PMN-MDSCs, transfer of MSU-induced PMN-MDSCs significantly increased serum uric acid content (Supplementary Figure 4B). These results suggest that MSU can induce substantial PMN-MDSC amplification and activation.

### Increased Production of IL-1β in Gout-Derived PMN-MDSCs

**Figure 5:**
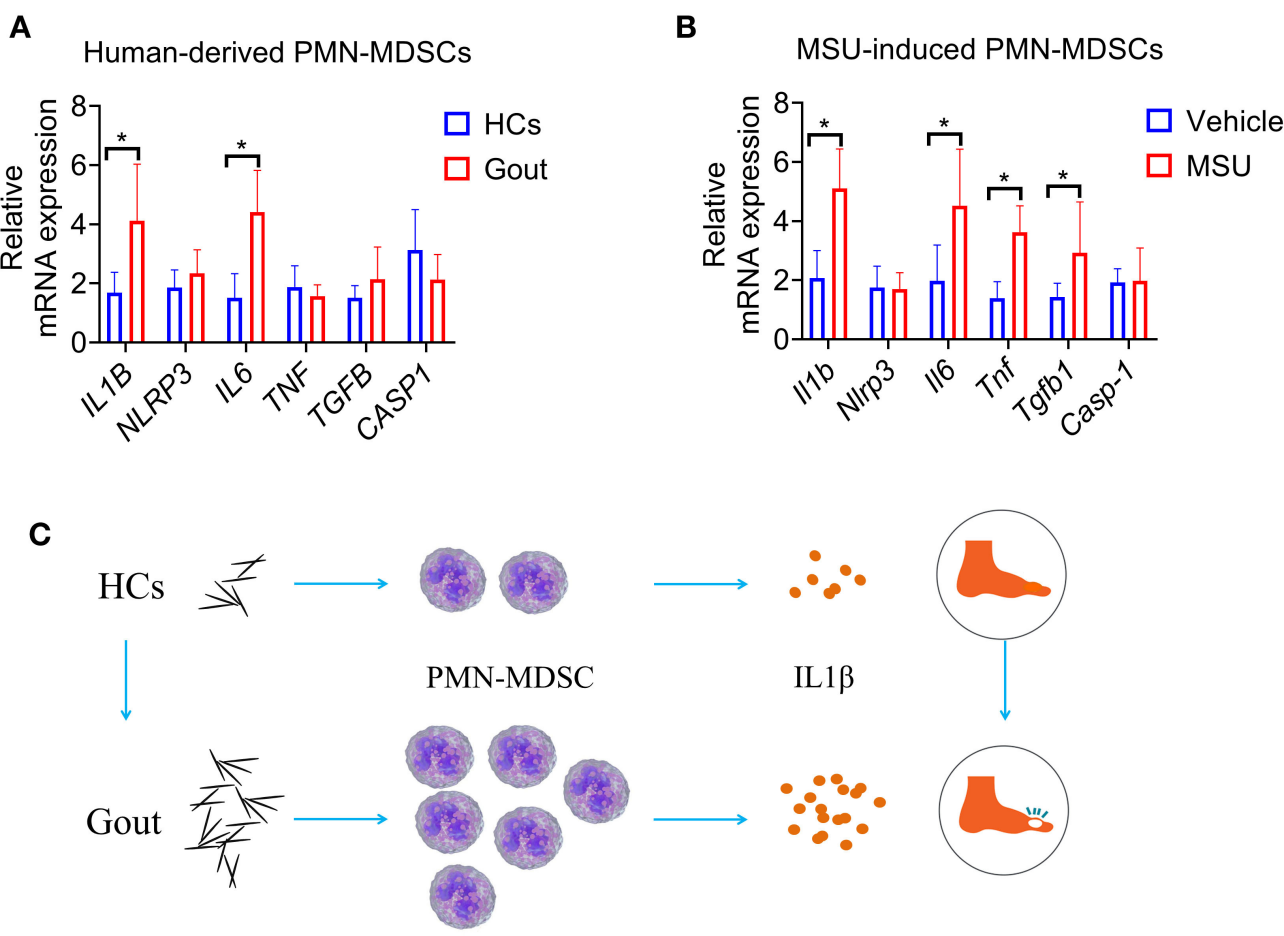
Increased production of IL-1β in gout-derived-PMN-MDSCs. **(A)** PMN-MDSCs sorted from HCs and patients with gout. qRT-PCR analysis for expression of inflammatory cytokines (*n* = 5). **(B)** qRT-PCR analysis for expression of inflammatory cytokines from MSU induced PMN-MDSCs (*n* = 5). **(C)** Study schematic diagram. Data are representative of two independent experiments. Error bars represent mean ± SD; ^*^P < 0.05.

To clarify the mechanism of PMN-MDSCs in regulating gout inflammation, PMN-MDSCs from healthy donors or patients with gout were sorted, and qRT-PCR was performed to quantify the levels of various inflammatory cytokines. Results showed that IL6 and IL-1β were significantly increased in patients with gout-derived-PMN-MDSCs compared to the control derived-PMN-MDSCs (Figure 5A). In addition, MSU induced a significant increase in the levels of inflammatory factors in WT mouse-derived-PMN-MDSCs, such as IL-6, IL-1β, TNF-a, and transforming growth factor (TGF)-β (Figure 5B). Among these, the levels of IL-1β were found to be the most significantly increased. Previous studies have demonstrated that IL-1β is an important cytokine that mediates the inflammatory response to gout (4). Considering that MDSCs were primarily responsible for the production of IL-1β, these results show that MSU can induce MDSCs to enhance the IL-1β-mediated inflammatory response (Figure 5C).

## Discussion

MDSCs are key regulators of various of inflammatory and autoimmune diseases and have prospective clinical applications, as well as implications for basic research (30, 31). Previous studies have shown that the frequency of MDSCs is elevated in mouse models of various autoimmune diseases, including multiple sclerosis, autoimmune hepatitis, inflammatory bowel disease, systemic lupus erythematosus, and RA, meanwhile the activity of MDSCs is associated with disease activity and progression (12). Specifically, an increasing in MDSCs is associated with the severity of joint damage in mouse models of experimental autoimmune arthritis, and in patients with RA (32, 33). Moreover, previous reports have shown that G-MDSCs (equivalent to our PMN-MDSCs) were increased in the peripheral blood of patients with gout, which is consistent with our study results (34). However, the mechanism by which PMN-MDSC regulates gout progression has not yet been characterized. Our study confirmed that PMN-MDSCs from patients with gout possess significant T cell inhibitory functions, while also demonstrated that MSU directly induces the expansion of PMN-MDSCs, and promotes their immunosuppressive functions. Furthermore, a potential mechanism for how PMN-MDSCs regulate the inflammatory response associated with gout was also elucidated, providing insights into potential cellular immunotherapeutic targets for gout.

Herein, the proportion of PMN-MDSCs in PBMCs was higher in patients with gout than in HCs, and the expansion of PMN-MDSCs, not M-MDSCs, was positively correlated with levels of uric acid and CRP. Previous studies have determined that CRP can directly induce MDSC expansion (35). For instance, during acute kidney injury in mice, CRP promotes MDSC generation, expansion, and renal infiltration, thereby driving the injury response (36). Meanwhile, MSU crystals induce inflammation by activating myeloid cells leading to inflammatory cytokine production and neutrophil activation (37). Hence, gout flares in patients may resemble septic arthritis (fever, high CRP) (38). A recent study reported the identification of CRP as a genuine MSU crystal recognition molecule, and speculated that CRP binding to MSU crystals modulates gout associated inflammation (39). Therefore, we suggest that CRP and MSU may exhibit synergistic activity in stimulating MDSC amplification.

We also confirmed that MSU can directly induce the expansion of PMN-MDSCs in humans and mice *in vitro*. These results confirm that the effect of urate deposition on inflammation may be mediated by MDSC expansion and activity. In addition, the levels of PMN-MDSCs in patients with gout were positively correlated with CRP levels, suggesting that PMN-MDSCs are related to acute inflammation in gout.

Numerous studies have shown that an increase in MDSCs promotes the differentiation of helper Th-17 cells and the progression of systemic lupus erythematosus and RA (23, 40). However, the precise mechanism underlying arthritis varies among different rheumatisms. A number of cytokines, such as TNF-α, IL-1, IL-6, IL-17, and matrix metalloproteinase 3 (MMP3), mediate the process of joint damage (33). Recent studies have also consistently identified a significant increase in MDSCs in the synovial fluid of patients with RA, as well as positive correlations between MDSCs and IL-17A levels. This previous research, as well as the results of the present study, support the important role of MDSCs in the development of inflammation via the induction of various cytokines or activation of immune cells. However, the precise mechanisms, and pathophysiological functions, of PMN-MDSCs in gout remain to be explored.

In summary, we provide evidence for the expansion of PMN-MDSCs in patients with gout and further show that these cells are related to disease activity and inflammation. Taken together, this data suggests that MDSCs are aberrantly produced in patients with gout and that PMN-MDSCs enhance the IL-1β-mediated gout inflammatory response. Further studies are required to clarify the precise functions and regulatory mechanisms of PMN-MDSCs in gout.

## Data Availability Statement

The data used to support the findings of this study are available from the corresponding author upon reasonable request.

## Ethics Statement

The studies involving human participants were reviewed and approved by the ethics review board of Guangdong Second Provincial General Hospital. Written, informed consent was provided by each participant and/or their legal guardian. The patients/participants provided their written informed consent to participate in this study.

## Author Contributions

LZ, SL, and YW: conceptualization. LZ: methodology, software, investigation, resources, writing-original draft preparation, and writing-review and editing. LZ and YW: validation. FL and YL: formal analysis. JZ: data curation. DC: visualization and supervision. DC and YL: project administration and funding acquisition. All authors contributed to the article and approved the submitted version.

## Conflict of Interest

The authors declare that the research was conducted in the absence of any commercial or financial relationships that could be construed as a potential conflict of interest.

## Abbreviations

CFSE: carboxyfluorescein succinimidyl amino ester
CRP: C-reactive protein
GM-CSF: granulocyte-macrophage colony stimulating factor
HCs: healthy controls
LOX-1: lectin-type oxidized low-density lipoprotein receptor-1
M-MDSCs: monocytic MDSCs
MSU: monosodium urate crystals
PMN-MDSCs: polymorphonuclear myeloid-derived suppressor cells
PBMCs: peripheral blood mononuclear cells.
